# Voluntary wheel running reduces tumor growth and increases capillarity in the heart during doxorubicin chemotherapy in a murine model of breast cancer

**DOI:** 10.3389/fphys.2024.1347347

**Published:** 2024-04-25

**Authors:** Tytti-Maria Uurasmaa, Chloé Ricardo, Anu Autio, Ilkka H. A. Heinonen, Helene Rundqvist, Katja Anttila

**Affiliations:** ^1^ Department of Biology, University of Turku, Turku, Finland; ^2^ Turku PET Centre, University of Turku, and Turku University Hospital, Turku, Finland; ^3^ Polytech Marseille, Aix-Marseille University, Marseille, France; ^4^ Department of Laboratory Medicine, Karolinska Institute, Stockholm, Sweden

**Keywords:** CS, LDH, VEGF, HIF, adjuvant therapy, cancer treatment, cardiotoxicity, exercise

## Abstract

**Introduction:** The possible beneficial effects of physical activity during doxorubicin treatment of breast cancer need further investigation as many of the existing studies have been done on non-tumor-bearing models. Therefore, in this study, we aim to assess whether short-term voluntary wheel-running exercise during doxorubicin treatment of breast cancer-bearing mice could induce beneficial cardiac effects and enhance chemotherapy efficacy.

**Methods:** Murine breast cancer I3TC cells were inoculated subcutaneously to the flank of female FVB mice (*n* = 16) that were divided into exercised and non-exercised groups. Two weeks later, doxorubicin treatment was started via intraperitoneal administration (5 mg/kg weekly for 3 weeks). Organs were harvested a day after the last dose.

**Results:** The tumor volume over time was significantly different between the groups, with the exercising group having lower tumor volumes. The exercised group had increased body weight gain, tumor apoptosis, capillaries per cardiomyocytes, and cardiac lactate dehydrogenase activity compared to the unexercised group, but tumor blood vessel density and maturation and tumor and cardiac HIF1-α and VEGF-A levels did not differ from those of the non-exercised group.

**Discussion:** We conclude that even short-term light exercise such as voluntary wheel running exercise can decrease the subcutaneous mammary tumor growth, possibly via increased tumor apoptosis. The increase in cardiac capillaries per cardiomyocytes may also have positive effects on cancer treatment outcomes.

## 1 Introduction

Breast cancer survivors have an increased risk of cardiovascular diseases (Afifi et al., 2020), and breast cancer patients treated with anthracyclines, such as doxorubicin, have a higher cumulative incidence of cardiomyopathy and/or heart failure than the general public and those who have not been treated with anthracyclines, although age and ethnicity might also play a role in the relative risk (Greenlee et al., 2022). Clinical studies have shown that exercise can have beneficial cardiac effects for breast cancer patients during and before anthracycline treatment (Sturgeon et al., 2014; Kirkham et al., 2017) and improve treatment outcomes (Courneya et al., 2014; Ansund et al., 2021), as well as decrease anthracycline-induced cardiovascular complications (Varghese et al., 2021). Preclinical studies have identified several underlying molecular mechanisms that are altered by physical exercise to induce cardioprotective effects against doxorubicin-induced cardiac damage, and it has also been shown that exercise could potentially enhance the chemotherapy effect on tumors via improved drug delivery (Betof et al., 2015). However, only few studies have investigated the cardiac benefits of exercise in breast cancer-bearing and doxorubicin-treated models, and studies on exercise and cardiac effects of doxorubicin often utilize healthy mice (Ghignatti et al., 2021). A recent, comprehensive review of the molecular mechanisms through which exercise mediates the cardioprotective effects against doxorubicin-induced cardiotoxicity also highlighted the need for more studies utilizing tumor-bearing models (Dozic et al., 2023). The lack of tumor burden can affect cardiac outcomes as tumors can affect the heart function and vasculature (Hoffman et al., 2021; da Costa et al., 2021; Tadic et al., 2018) and the animal’s capability for physical activity (Smeda et al., 2017). Breast cancer alone has been shown to induce weight loss, decrease cardiac expression of Mitofusin-1, which mediates mitochondrial fusion (Jafari et al., 2021), cause dysfunction of cardiac calcium handling (da Costa et al., 2021), and affect the cardiac capillary permeability (Hoffman et al., 2021). Therefore, there is a need for more studies on the cardiac effects of exercise interventions during doxorubicin treatment of breast cancer tumor-bearing models. Incorporation of the cancer condition in exercise studies will also improve the current understanding regarding the needed exercise prescription as the presence of cancer could, together with treatment, further blunt the benefits of exercise.

Doxorubicin can affect the heart function and induce cardiotoxicity via multiple mechanisms, but one of the main mechanisms is thought to be increased oxidative stress in the heart via increased formation of reactive oxygen species (Montalvo et al., 2020). Additionally, doxorubicin has been found to damage cardiac vasculature and decrease the cardiac capillary area (Räsänen et al., 2016; Hoffman et al., 2021), thus contributing to doxorubicin-induced cardiotoxicity (DIC) (Räsänen et al., 2016; Hoffman et al., 2021). So far, there seem to be relatively few studies on the effect of exercise interventions on doxorubicin-induced cardiotoxicity utilizing female mice (Ghignatti et al., 2021). Female mice have been shown to be less susceptible to doxorubicin-induced cardiotoxicity due to the protective effects of estrogen (Rattanasopa et al., 2019), but they have also been shown to be physically more active than male mice (Lightfoot et al., 2004). Therefore, there is also a need for more studies on the cardiac effects of exercise in doxorubicin-treated female mice. The few animal studies that have been done on the cardiac effects of exercise during doxorubicin treatment of breast cancer, similar to the studies without tumor burden, show that doxorubicin increases acutely cardiac apoptosis and double-stranded DNA breaks (Cheong et al., 2021) and induces cardiac atrophy, functional impairment, and cardiac accumulation of doxorubicin (Parry and Hayward, 2015; Wakefield et al., 2021). Of these effects, exercise can prevent or mitigate at least acute cardiac atrophy and functional impairment and the initial accumulation of doxorubicin in mammary tumor-bearing animals (Parry and Hayward, 2015; Wakefield et al., 2021). However, differing results have also been found. It has been shown that in the case of low-to-moderate intensity exercise performed concomitantly with treatment, exercise did not mitigate the reduction of cardiac ejection fraction in non-tumor-bearing male mice despite exercise mitigating cardiac atrophy and myocardial strain (Gomes-Santos et al., 2021a). Furthermore, there are dissimilar results regarding whether exercise can improve the doxorubicin efficiency in reducing the tumor volume (Parry and Hayward, 2015; Wakefield et al., 2021). Overall, preclinical studies mainly done using healthy animals suggest that exercise can help alleviate the cardiotoxic effects of doxorubicin (Dozic et al., 2023). To the best of our knowledge, no study has yet investigated whether exercise could improve cardiac capillarization or cardiac and tumor citrate synthase and lactate dehydrogenase activities in doxorubicin-treated mammary tumor-bearing animals. In healthy animals, exercise has been shown to improve cardiac capillarization (White et al., 1998; Laughlin et al., 2012; Machado et al., 2017) and induce physiological cardiac hypertrophy (Allen et al., 2001).

The current study investigates exercise effects in a mouse breast cancer model treated with doxorubicin. All murine cancer models are only able to mimic certain aspects of the equivalent human cancer, and thus, studies using multiple models are needed to be able to eventually draw conclusions that are better translatable to humans. The pros and cons of different murine tumor models have been previously reviewed thoroughly (Zeng et al., 2020).

The few previous studies with exercise intervention of doxorubicin-treated breast cancer-bearing rodent models have utilized both subcutaneous and orthotopically implanted models (Parry and Hayward, 2015; Wakefield et al., 2021). In a previous study, a subcutaneous breast cancer model was established using adenocarcinoma with high metastatic potential (Parry and Hayward, 2015). The cell line utilized in the current study is a rapidly growing, subcutaneous adenocarcinoma. The subcutaneous tumor models can mimic the growth of a tumor that has spread to nearby regions from the tissue of origin; although, as with all implanted tumors, they skip the initiation phases of tumor formation and also the growing phase within the tissue of origin. Previously, effects of exercise have been studied in doxorubicin-treated tumor-bearing animals using exercise preconditioning, thus comparing the study conditions to a situation of physically active individuals getting cancer and being treated for it (Parry and Hayward, 2015). The current study will aim to investigate a situation where exercise is started upon cancer initiation, which better resembles a situation where exercise is started upon diagnosis. One of the previous studies using orthotopically implanted mouse breast cancer has studied the effects of exercise and doxorubicin on tumor growth in older mice; thus, their results apply better to the older population (Wakefield et al., 2021). However, they studied the cardiac effects in a non-tumor-bearing model. The orthotopic models better mimic primary tumor growth in the tissue of origin, although they skip tumor initiation and risk the tumor growth affecting the running activity due to the location of the tumor and mechanical irritation. Additionally, all syngeneic mouse tumors have the drawback of being non-human in origin, but they allow the use of immunocompetent animals, which are more translatable to human tumors.

The first aim of the current study was, therefore, to assess the cardiac effects of short-term voluntary wheel running in female mice during doxorubicin treatment of subcutaneous mammary tumors with a dosage based on previous studies (Podyacheva et al., 2021). Along these lines, we aimed to compare the cardiac capillary density, factors regulating angiogenesis, the markers of oxidative stress status, the activity of anti-oxidative enzyme catalase, and the activity of metabolic enzymes of aerobic and anaerobic energy production between exercising and non-exercising mice with breast cancer tumor receiving doxorubicin treatment.

The second aim of our study was to assess whether short-term voluntary wheel-running exercise during doxorubicin treatment could reduce the tumor size compared to doxorubicin treatment alone (Betof et al., 2015). The apoptosis and tumor cell proliferation markers were histologically analyzed from the tumors to measure the changes in tumor growth in response to treatment and exercise. Since exercise can potentially affect tumor growth via its effects on tumor vascularization and, thus, drug delivery, we set out to also analyze the tumor blood vessel density, the expression of factors regulating angiogenesis, and the changes in the aerobic and anaerobic metabolism. Additionally, since tumor vasculature can be structurally and functionally abnormal, we analyzed tumor blood vessel maturation by measuring the pericyte coverage of the blood vessels.

## 2 Materials and methods

### 2.1 Cell culture

Mammary tumor cells of the I3TC cell line were derived from the mammary tumors of transgenic mice (FVB) expressing mammary tumor virus–polyoma middle tumor-antigen (MMTV-PyMT) according to a previous study (Weiland et al., 2012). In this previous study, the cells were characterized and found to form poorly differentiated tumors, which had not reached independence from factors secreted by tumor or cancer-associated fibroblasts. The tumor cells of the MMTV-PYMT transgenic model have been further characterized in detail previously (Lin et al., 2003). The authors found that the tumors at the advanced stage of malignant transition lose virtually all progesterone receptors and that in within 80% of the animals, 90% of the tumor cells are ER-α-negative. The cancer cell line was derived from these tumors, thus likely having a mix of cells with these qualities. Due to their tendency to progress similarly to human cancer and their fast-multiplying potential, the current cell line implanted subcutaneously mimics the aggressive growth of a tumor that has spread to nearby regions from the tissue of origin (Supplementary Figure S1). This type of aggressively growing tumor is also a realistic target for chemotherapy treatment. The cells were frozen prior to experiments, stored in liquid nitrogen, and obtained from the same batch of cells used previously in another study (Rundqvist et al., 2020). Cells were thawed 6 days before inoculation and transferred to a warm growth media containing filtered sterilized (0.22 µm) Dulbecco’s modified Eagle's medium (Gibco, REF14190-094) supplemented with 10% fetal calf plasma (Thermo Fisher Scientific, 10,270–106) and 1% penicillin and streptomycin (Thermo Fisher Scientific, 15,140,122). Cells were pelleted and re-suspended in fresh growth media and cultured at 37°C and 5% CO_2_. At passage 2 and a confluency of 90%, the cells were collected for inoculation. Cells were detached with TrypLE™ (Gibco, REF 12605–028) and incubated at 37 °C for 5–10 min, after which trypsin was inactivated with a growth medium, and cells were spun down and re-suspended in Dulbecco’s phosphate-buffered saline (Gibco, REF 14190–094).

### 2.2 Animals

The I3TC mouse mammary tumor cells (n = 16, 2×10^6^ cells/mouse) were inoculated subcutaneously in the middle of the flank of female FVB/NRj mice (Janvier Labs, age: 8 weeks, average mass: 22.1 g, and n = 16). Being an explorative study, power calculation for choosing the n-number could not be performed, but according to previous studies, the n = 5–6/group has been sufficient for detecting exercise effects on different cancer models (Schadler et al., 2016; Gomes-Santos et al., 2021b). Ectopic implantation of tumor cells was used because orthotopic implantation might intervene with the running ability of the mice due to the tumor location underneath the animal when the tumors grow to a larger size. Three days after inoculation, the mice were divided randomly into the exercising (n = 8) and non-exercising (n = 8) groups and housed in pairs (n = 2 mice/cage; 1 wheel/cage) in rat IVC cages to reduce their stress from being alone. The exercising group was housed with wireless low-profile running wheels (Med Associates Inc ENV-044, St Albans, United States), and the non-exercising control group was housed with the same type of wheels with stoppers to prevent the wheels from spinning. Med Associates Inc. wheel manager software and wheel analysis software (SOF-860 and SOF-861) were used to record the spins of the wheels. All mice were housed in the central animal laboratory of Karolinska Institute under controlled conditions and kept under a 12-h light–dark cycle with *ad libitum* access to water and the same type of standard pellet feed. The food intake was not monitored throughout the study.

A week after tumor cell inoculation, tumor growth monitoring was started by measuring the tumor size with calipers at two dimensions (length and width, with the volume calculated with a formula of length*width*width*3.14/6) three times a week throughout the study period (Supplementary Table S1). Additional tumor growth data from randomly selected female FVB mice (n = 5, Inotiv) without doxorubicin treatment and without exercise are shown in Supplementary Figure S1 for comparing the tumor growths. These mice were inoculated with the same number of I3TC mouse mammary tumor cells at the University of Turku at a later time point. At the end of the study, the tumor weights were estimated for the doxorubicin-treated groups using a linear function determined from the relationship between the tumor volume and weight in this tumor model (Supplementary Figure S2). The doxorubicin treatment plan was chosen according to previous studies. The dosages of doxorubicin in cardiotoxicity studies have been reviewed in mice, and they range from 2 to 8 mg/kg with a cumulative dose ranging between 10 and 40 mg/kg (Podyacheva et al., 2021). Those studies that have been carried out using female mice have used total cumulative doses of 15–24 mg/kg (Podyacheva et al., 2021). Accordingly, we chose to use a total cumulative dose of 15 mg/kg.

The mice received doxorubicin (Merck, 324,380) diluted in PBS weekly (5 mg kg^-1^) via intraperitoneal injections starting 14 days after the tumor inoculation. Mice were treated for 3 weeks or until their tumor volume reached the end point of >1 cm^3^, after which they were euthanized using CO_2_ (with a volume displacement rate of 20% per minute), and their organs were harvested. Mice were also euthanized before the endpoint if the tumors ulcerated or if the animals had significant weight loss. Only one animal had to be euthanized earlier due to tumor size (unexercised group, day = 21) and one due to tumor ulceration (exercised group, day = 21). There was no evidence of diarrhea in the animals throughout the study. The animals were handled according to the institutional animal‐care policies of the Karolinska Institute. The experiments were conducted under the ethical license N101-16 according to Swedish legislation, and the ethical license was approved by the Swedish Agricultural Agency’s regional animal testing committee of Stockholm.

### 2.3 Immunohistochemistry

Transversal sections of the hearts and half of the tumors were treated for 24 h in 4% formaldehyde, after which they were transferred into 70% ethanol. The tissues were dehydrated in an increasing alcohol series and embedded in paraffin. Heart sections of 5 µm and tumor sections of 4 µm were rehydrated in a decreasing alcohol series, the antigen was retrieved, and the endogenous peroxide activity was quenched if needed (Supplementary Table S2). The unspecific binding sites were blocked with 1% bovine serum albumin (BSA) in PBS before antibody labeling with primary and secondary antibodies. The heart and tumor blood vessels were detected with podocalyxin (1:300, R&D systems AF1556), tumor blood vessel maturation/pericyte coverage was detected with the podocalyxin and Anti-Actin α-Smooth Muscle-Cy3™ antibody (α-SMA, ex. max 555 nm, em. max 569 nm 1:100, Sigma-Aldrich C6198, clone 1A4), tumor apoptosis was detected with cleaved caspase 3 antibody (1:200, R&D Systems AF835), and tumor proliferation was detected with the ki67 antibody (1:200 Sigma-Aldrich ab9260). The concentration and specificity of α-SMA, cleaved caspase 3, and ki67 are based on previous studies (Yin et al., 2015; Ouarné et al., 2018; Wijler et al., 2022). The details of the staining protocol are specified in Supplementary Table S2. Anti-rabbit or anti-goat biotinylated secondary antibodies were used (1:500), except for blood vessel maturation stain, for which the fluorescent anti-goat AlFl.488 (ex. max 499, em. max 520 nm 1:100, LifeTech A11055) secondary antibody was used. Sections stained with fluorescent dye were sealed with the ProLong™ Gold Antifade reagent with DAPI (Invitrogen P36931), and the DAB-stained sections were counterstained with Mayer’s hematoxylin (Sigma-Aldrich), sealed using aqueous mounting medium (Kaiser’s glycerol gelatin, 108635 Sigma-Aldrich), and further sealed with nail polish. Heart and tumor sections were also stained with hematoxylin–eosin stain for structural reference (Supplementary Figures S3–S4).

All the slides were imaged blinded using a Nikon Eclipse microscope at a magnification of ×20 (no filter or filters DAPI, FITC, and TRITC), Nikon pE-300 ultra camera, and NIS-Elements AR software. All the images were analyzed using ImageJ 1.53c. The heart capillary and tumor blood vessel densities were calculated manually from four to five different areas and a minimum total area of 0.1 and 1.1 mm^2^ of heart and tumor tissues, respectively. Tumor staining was done on transversal sections from the middle of the tumor. Of these sections, the non-necrotic regions were used for analysis, and regions from the outermost regions of the tumor periphery were avoided. If necrosis was present in the tumor, it was usually located in the middle of the tumor, and this area was usually lost during the processing of the sections as it was fairly soft/nearly liquid in consistency. The tumor blood vessel maturation was analyzed manually in a blinded manner by first removing the background from images taken with the FITCH filter using the script provided in Supplementary Data S1, after which the blood vessels were manually counted from four to five different areas and a minimum total area of 0.7 mm^2^ of tissue. The regions of interest indicating the blood vessels were overlaid with the TRITC filter image, from which the background was subtracted using the retinex function in ImageJ; this image was used to count the α-SMA-positive blood vessels. To reduce risk of bias, cas3- and ki67-positive cells per area of tissue (min 0.1 mm^2^) were detected using custom-made scripts provided in Supplementary Data S1. These scripts applied a threshold for positive cells and used particle count to calculate the positive cells. Color deconvolution 2 was used in the script (Ruifrok and Johnston, 2001; Landini et al., 2021). Outlier values were manually checked and recalculated if needed.

### 2.4 Western blot

The frozen heart left ventricles and the mammary tumors were crushed, and tissue pieces were weighed, homogenized, and processed for Western blot, as described previously (Uurasmaa et al., 2021). The protocol and products used for the tissue preparation are specified in detail in Supplementary Table S3. For the hearts and tumors, 20 and 30 μg of protein were separated for antibody labeling, respectively, using the TGX stain-free fast cast acrylamide kit 12% (Bio-Rad). The total protein amount per lane was determined by imaging gels with the ChemiDoc™MP imaging system. Thereafter, the proteins were transferred onto the Amersham™ Protran™ nitrocellulose blotting membrane (0.45 µm, GE Healthcare Life Science, Germany). The membranes were cut and washed, after which unspecific binding sites were blocked for 1 hour in room temperature in 5% fat-free milk diluted in TRIS-buffered saline (TBS). Antibody staining for HIF1-α and VEGF-A (Rabbit monoclonal, Abcam, ab2185, & ab214424) was done overnight at +4 °C in the primary antibody dilution of 1:2,000 (1:1000 for heart VEGF-A) diluted in a blocking solution with 0.1% Tween-20. Secondary antibody staining (IRDye^®^ 800CW 926–32211, goat anti-rabbit, LI-COR) was done in 1:5,000 dilution in a blocking solution with Tween for 1 h at room temperature. The membranes were imaged using the ChemiDoc™MP imaging system (Bio-Rad), and the intensity of the bands was calculated using Image Lab 6.0 (Bio-Rad). The intensities of protein bands of interest were divided with the overall protein band intensity that was calculated from the gel images; the exemplary full Western blot membrane and gel images are provided in Supplementary Figure S5.

### 2.5 Enzymatic activity and oxidative stress measurements

The heart apex and part of the left ventricle were pooled and weighed, and the same was done for the remaining tumor pieces. The tissues were homogenized in 1:10 mg µL-1 of the homogenization solution (50 mM Hepes, 1 mM EDTA, 0.1% Triton-X, pH 7.4), as stated in Supplementary Table S3. The tumor and heart homogenates were aliquoted for the measurement of citrate synthase (CS) and lactate dehydrogenase (LDH) activities and diluted in Tris 50 mM pH 8.0 and Tris 50 mM pH 7.4, respectively. The heart homogenates were further aliquoted for the measurement of lipid peroxidation, and the rest of the homogenate was centrifuged at 4 °C 10,000 G for 10 min, after which the supernatant was snap frozen for the measurement of protein concentration (Bicinchoninic acid assay, Pierce^®^, Thermo Fisher Scientific, United States) and catalase (CAT) activity. The measurements for the CS-activity, LDH-activity, CAT-activity, and the amount of lipid hydroperoxides (indicative of lipid peroxidation) were performed according to our previous study (Uurasmaa et al., 2021). There was not enough tissue to measure the CS-activity and LDH-activity from all the tumors; thus, these measurements have a lower n-number.

### 2.6 Statistical analysis

All data were tested for normal distribution and equality of variance using the Shapiro–Wilk and Brown–Forsythe tests, respectively. Outliers were defined as values that differed from group average ≥ 2 × SD. All analyses were done on data with the outliers excluded, except for the tumor volume data, where no exclusions were made. For the molecular measurements (Western blot, histology, and enzymatic analyzes), the decision to remove outliers was taken because they may also be indicative of erroneous values caused by methodological failures rather than true physiological outliers. All comparisons between the two groups were carried out using Student’s t-test, with Welch’s t-test being used if the data did not have equal variance. The change in tumor volume over time was compared between the two groups using the linear model on repeated measures, with the time and test group as the fixed factors. The animals that were euthanized earlier due to tumor growth were included in all the time points using their last measured tumor volume due to their exclusion significantly affecting the average tumor volume between the time points. The linear model should be robust enough to be used on the tumor volume data that do not follow normal distribution. *p* values <0.05 were considered significant, and test power was at minimum 0.8, unless otherwise stated.

The running activity of the exercise group was calculated using the spins and the diameter of the wheel. To obtain the running activity of a single mouse, the wheel readings were divided by two since the mice shared a wheel and were assumed to use the wheel equally. It must be noted that this underestimates the mouse running activity as the mice have been witnessed to use the wheel simultaneously as well. The running activity between the groups and between three different timeframes was analyzed using one-way ANOVA. The three different timeframes were the following: prior treatment (week 1–2), after one dose (week 3), and after more than 1 dose (week 4). All data values are average ± standard error, unless otherwise stated, and SigmaPlot 14 or SPSS 27 was used for all the statistical tests.

## 3 Results

**FIGURE 1:**
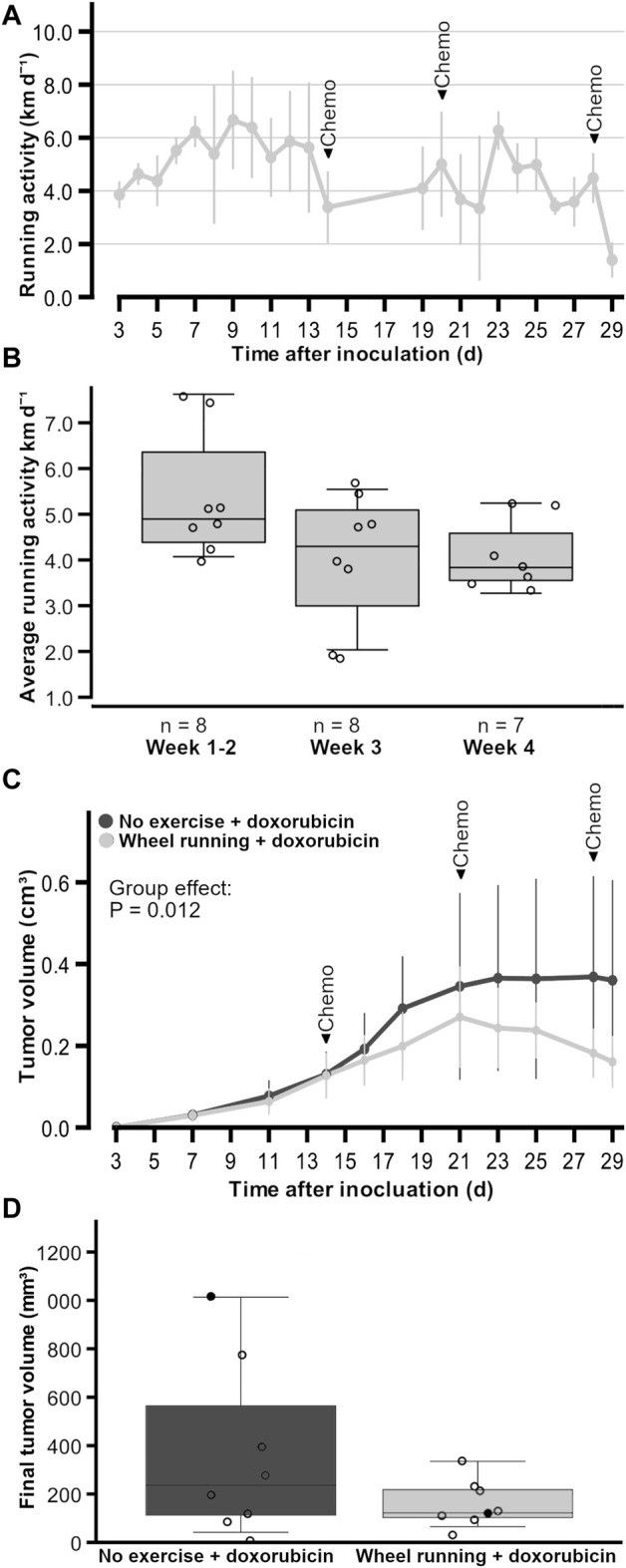
Running activity and tumor growth of female FVB mice that had subcutaneous mammary tumors (13TC) and received doxorubicin (5 mg/kg/week). **(A)** Running activity throughout the study period. **(B)** Average running activity during three different timeframes. **(C)** Tumor volume throughout the study period. **(D)** Final tumor volumes with individual values shown. **(D)** Black data points indicate the animals that had to be euthanized earlier due to tumor volume or wound. The timeframes for running activity are prior to chemotherapy (week 1–2), after one dose of chemotherapy (week 2), and after more than one dose of chemotherapy (week 3). The exercise group performed voluntary running wheel exercise, while the control group is the non-exercised group. Holm–Sidak *post hoc* *p < 0.05. The group n-numbers are indicated within each figure, and the *p*-value in **(C)** indicates the statistical difference between groups tested with the linear model. **(A, C)** are average ± standard error x 2.

The exercising period lasted 3.7 ± 0.1 weeks, and the mice ran on average 4.7 ± 0.6 km/d (Figure 1A). The running activity of the exercising mice did not change over the different timeframes (*p* = 0.085, power <0.8), although there was a trend of the running activity decreasing with the start of chemotherapy (Figure 1B). There was barely any bodyweight gain in the non-exercising control group animals (0.1 ± 0.8 g) during the study, while the exercising group had significantly more bodyweight gain (1.4 ± 0.6 g, *p* = 0.003), although the bodyweights without tumor weights did not differ between the non-exercising and exercising groups by the end of the study (22.6 ± 1.7 g, 23.0 ± 1.1 g, respectively, *p* = 0.642).

All tumors of the doxorubicin-treated animals became palpable (∼1 mm in diameter) 3 days following the inoculation. The final tumor volumes of the animals did not statistically significantly differ between the exercising and non-exercising control groups (160.7 ± 30 mm^3^, 360 ± 115 mm^3^, respectively, *p* = 0.279, Figures 1C and D), although the average tumor volume in the exercise group was only 44% of the average tumor volume in the unexercised group. Similarly, the estimated tumor weights did not differ between the exercising and non-exercising control groups (0.2 ± 0.1 g, 0.5 ± 0.4 g, respectively, *p* = 0.279). However, despite no differences in final tumor volumes between the test groups, when the linear model on repeated measures (AIC = 1631.479, Model Intercept *p* < 0.001, F = 160,932) was used and the tumor volumes were compared throughout time, there was a significant fixed factor effect of the group (*p* = 0.012, F = 6.662), with the exercise group having smaller tumor volumes (Figure 1C). There was no interaction between the statistical factors time and group (*p* = 0.550, F = 0.874). These data did not follow normal distribution, which was the reason the more robust linear model was used instead of two-way RM ANOVA. The individual tumor growths are provided in Supplementary Figure S1 for doxorubicin-treated groups and the untreated group inoculated at a later time point in a different facility. From the individual tumor growths, it is apparent how these untreated tumors reached end point criteria much faster than the treated tumors, and Supplementary Figure S1A shows that, on average, the untreated animals had larger tumor volumes over time.

**FIGURE 2:**
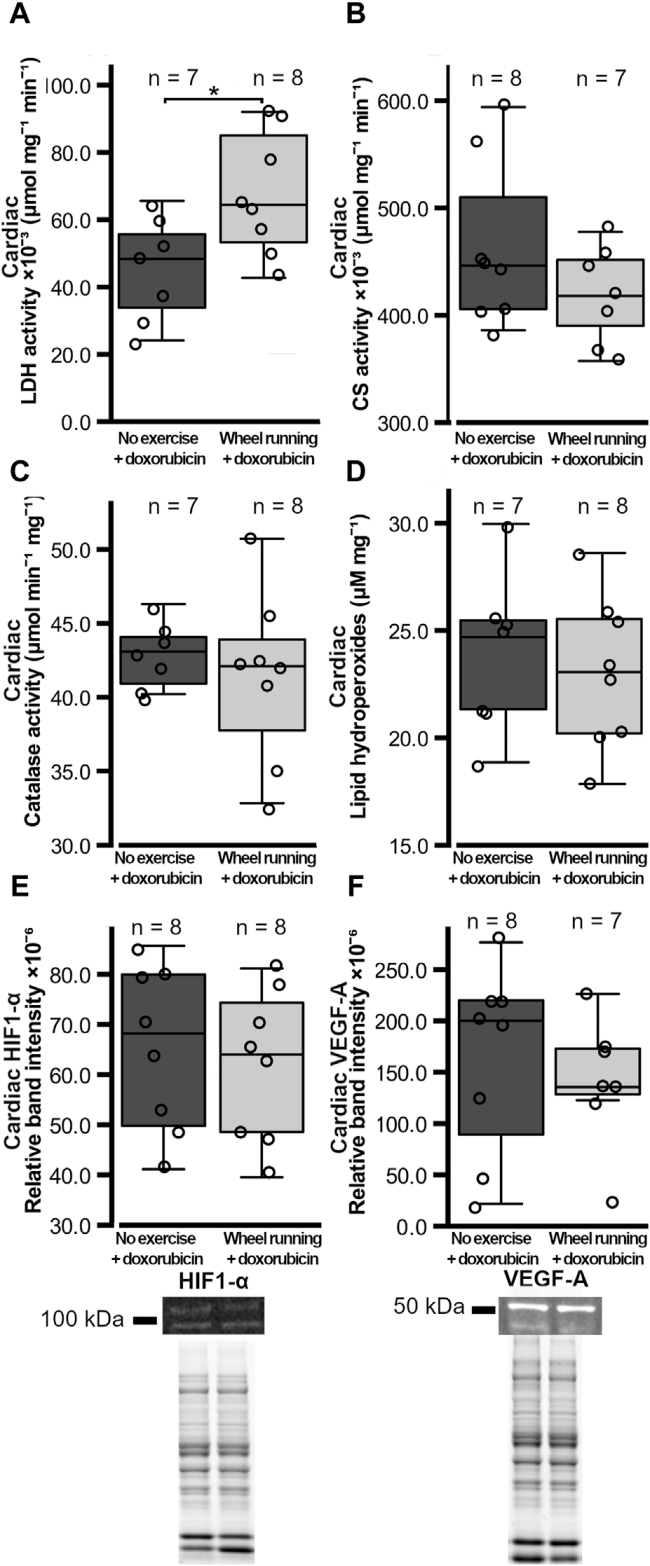
Heart metabolic enzyme activities, cardiac oxidative stress markers, and HIF1-α VEGF-A levels in female FVB mice that had subcutaneous mammary tumors (13TC) and received doxorubicin (5 mg/kg/week). **(A)** Heart LDH-activity, **(B)** CS-activity, **(C)** catalase activity, **(D)** lipid peroxidation, and **(E)** cardiac HIF1-α and **(F)** VEGF-A levels are shown with the group n-numbers in the figures. The exemplary Western blot band images and overall protein band images are shown below the respective graphs. The exercise group performed voluntary running wheel exercise, while the control group is the non-exercised group. Student’s t-test *p < 0.05.

The cardiac LDH activity was significantly higher (t = 2.536, *p* = 0.0249) in the exercising group when compared to the non-exercising control group (Figure 2A). However, the cardiac CS activity did not differ between the groups (*p* = 0.210, Figure 2B). In addition, the cardiac catalase activity and the level of cardiac lipid hydroperoxides did not differ between the groups (*p* = 0.547, *p* = 0.650, respectively, Figures 2C and D). The protein levels of cardiac HIF1-α and VEGF-A were also similar between the groups (*p* = 0.663, *p* = 0.566, respectively, Figures 2E and F).

**FIGURE 3:**
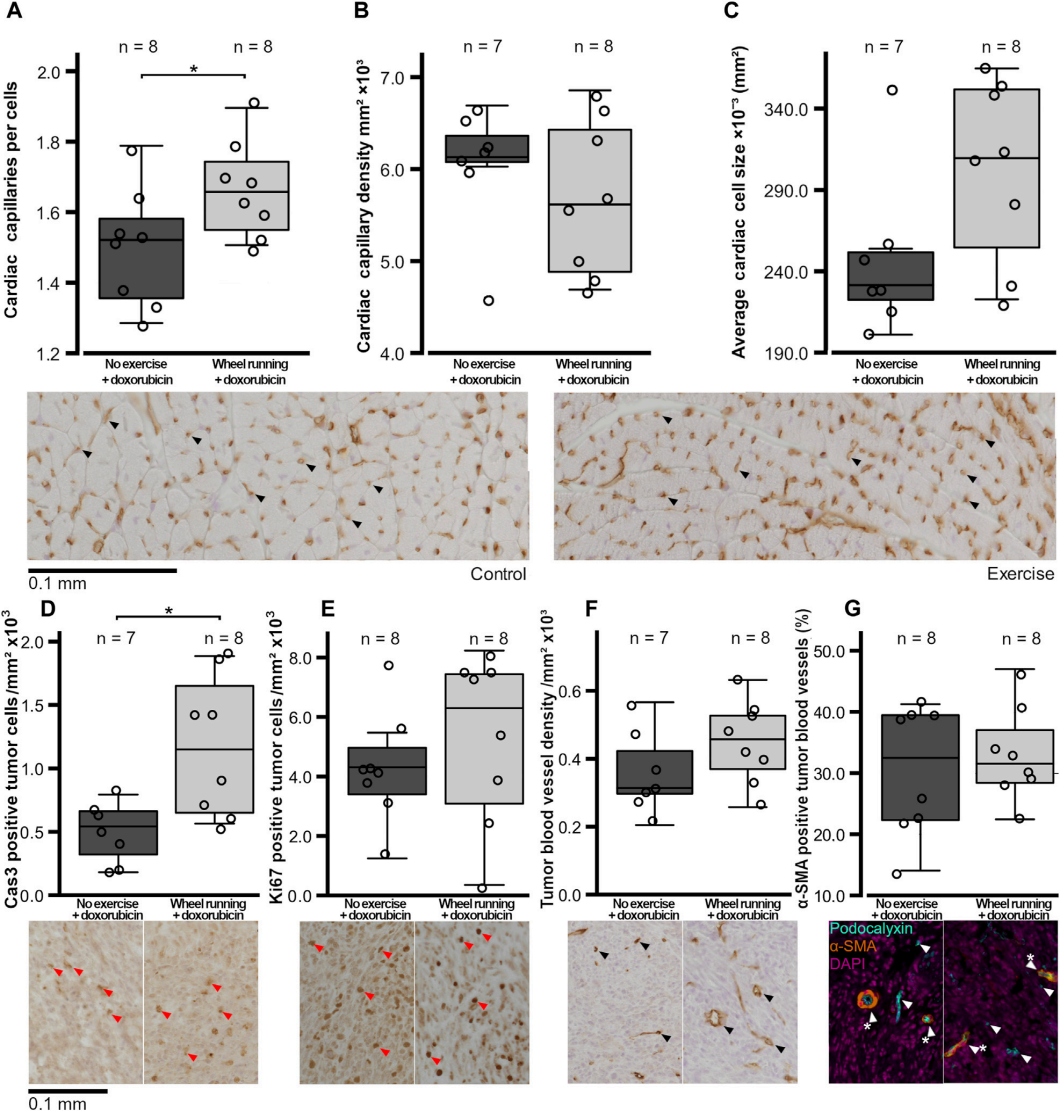
Histological variables of the hearts and tumors of the female FVB mice that had subcutaneous mammary tumors and received doxorubicin (5 mg/kg/week). **(A)** Number of capillaries per cells in the heart, **(B)** heart capillary density, **(C)** average cross-sectional cardiomyocyte size, **(D)** number of cas3-positive tumor cells/mm^2^, **(E)** number ki67-positive tumor cells/mm^2^, **(F)** tumor blood vessel density, and **(G)** percentage of α-SMA-positive tumor blood vessels are shown with the n-numbers marked in the figures. The exercise group performed voluntary running wheel exercise, while the control group is the non-exercised group. The exemplary histological images or fluorescent labeled composite images are shown below figures, with the blood vessels indicated by black or white arrows, the α-SMA-positive vessels being indicated by a white star, and the DAB-positive cells being indicated by red arrows. All the histological images are imaged at ×20 magnification, and the image contrast may have been edited equally per stain for visibility in the figures, but unedited images were used for analysis. Student’s t-test *p < 0.05.

The number of cardiac capillaries per cells was significantly higher in the exercising group compared to that of the non-exercising control group (t = 2.183, *p* = 0.0466, Figure 3A), although there was no significant difference in the cardiac capillary density between the two groups (*p* = 0.368, Figure 3B). There was also a trend of the average cardiac myofibril cross-sectional area being larger in the hearts of animals in the exercised group compared to the non-exercised control animals, although this difference was not quite statistically significant (t = 2.004, *p* = 0.0664, Figure 3C).

**FIGURE 4:**
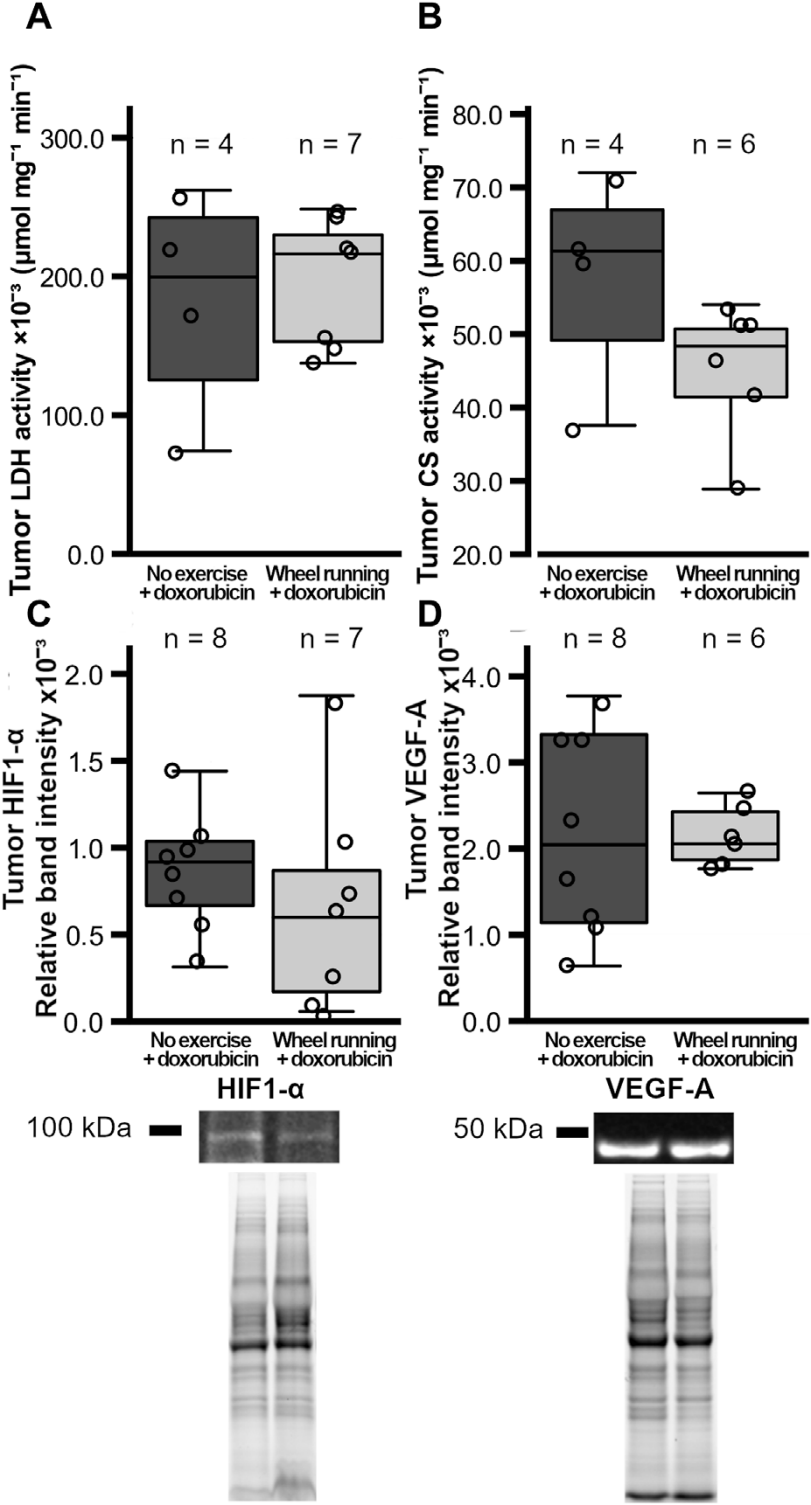
Tumor metabolic enzyme activities and HIF1-α VEGF-A levels in female FVB mice that had subcutaneous mammary tumors (13TC) and received doxorubicin (5 mg/kg/week). **(A)** Tumor LDH-activity, **(B)** CS-activity, and **(C)** the tumor HIF1-α and **(D)** VEGF-A levels are shown with the group n-numbers in the figures. The exercise group performed voluntary running wheel exercise, while the control group is the non-exercised group.

The number of cells/mm^2^ positive for the marker of apoptosis, cleaved caspase 3, was significantly higher in the tumors of the exercising animals (t = 3.131, *p* = 0,0110, Figure 3D), whereas the number of tumor cells positive for the proliferation marker ki67/mm^2^ was similar between the two groups (*p* = 0.434, Figure 3E). The tumor blood vessel density was also similar between the two test groups (*p* = 0.176, Figure 3F), and the percentage of α-SMA-positive blood vessels in the tumors did not differ between the groups (*p* = 0.593, Figure 3G). Both the tumor LDH and CS activities were similar between the groups (*p* = 0.765, *p* = 0.123, respectively, Figures 4A,B). Similarly, the protein levels of tumor HIF1-α and VEGF-A did not differ between the groups (*p* = 0.420, *p* = 0.927, respectively, Figures 4C and D).

## 4 Discussion

The first aim of the study was to determine the cardiac effects of short-term running wheel exercise during doxorubicin treatment of subcutaneous mammary tumors in mice. The results revealed that even short-term exercise can be beneficial for the heart energy metabolism, at least in regard to LDH activity and capillary number per cardiomyocytes, which could make them more resistant to doxorubicin-induced cardiac damage. Additionally, exercise had a slight beneficial effect on animal bodyweight change, considering that doxorubicin treatment is known to often cause weight loss. The second aim of the study was to assess whether short-term voluntary wheel-running exercise and chemotherapy treatment together could enhance the tumor size reduction. The results showed that even short-term voluntary running wheel exercise during treatment might increase the treatment efficacy of doxorubicin on subcutaneous mammary tumors via increased apoptosis, although the exact mechanism needs further investigation.

### 4.1 Mouse physical activity and the cardiac alterations

The weekly intraperitoneal administration of 5 mg/kg of doxorubicin did not significantly reduce the running activity of mice. At this stage of the treatment regimen, there was also no significant bodyweight loss, which is often seen alongside the cardiotoxic effects of doxorubicin (Sahu et al., 2016; Pillai et al., 2017). However, there was a significantly less bodyweight increase in the non-exercising animals compared to the exercising animals, and thus, we cannot rule out cardiac alterations associated with less severe or mild toxicities without more experiments with untreated or healthy controls. The FVB/N mice are known to naturally gain weight at this age even when on low-fat diets (Luijten et al., 2013), and although the non-exercising animals did not, on average, lose weight, they had little, if any, bodyweight increase, which suggests that doxorubicin might have blunted the bodyweight gain. Therefore, the bodyweight gain in exercising animals could be seen as a positive effect. We would also like to acknowledge that exercise could have altered food intake, especially considering that doxorubicin is known to decrease appetite (O’Keefe et al., 1993), and this could affect our results. However, these effects on appetite, although indirect, would still be exercise-induced. When compared to previously published studies, our mice ran a similar distance, 4.1–5.3 km, compared to the 3.5–4.5 km per day shown before (Lerman et al., 2002). This is important since mice staying active would allow us to answer the first question of the experiment, i.e., could physical exercise induce beneficial cardiac alteration during doxorubicin treatment of mammary tumor-bearing mice. The exercise for nearly 4 weeks did not induce a significant increase in capillary density in the left ventricle. This may partially be due to the low n-number, which may mean that smaller differences between groups may go undetected. However, there was a trend of increased cardiomyocyte size and a significantly increased capillary per myocyte ratio, which suggest that this level of exercise may have some cardiovascular benefits for subcutaneous mammary tumor-bearing mice receiving doxorubicin treatment. The increased number of capillaries per cardiomyocytes can be considered beneficial for doxorubicin-treated animals since doxorubicin has been shown to reduce the cardiac capillary area and increase cardiac capillary permeability, which can further contribute to doxorubicin-induced cardiotoxicity (Räsänen et al., 2016; Hoffman et al., 2021). Previously, it has been shown that voluntary wheel running of 6.8 km/day in healthy male C57/Bl6 mice for 4 weeks induces cardiac hypertrophy (Allen et al., 2001) and that treadmill running exercise dose dependently increases cardiac capillary density in rats (Machado et al., 2017). However, with swines, it has been shown that exercise training increases cardiac capillary density only initially, until cardiac hypertrophy exceeds the exercise-induced angiogenesis (White et al., 1998; Laughlin et al., 2012). Therefore, it may be that in this study, the exercise-induced increase in capillary density is partially masked by the increased cardiomyocyte size, although not completely, as evidenced by the still significantly increased capillary to cardiomyocyte ratio. It is possible that a greater intensity and longer duration of exercise may yield even greater benefits from exercise performed concomitantly with chemotherapeutic treatment. Further experiments with control groups are also needed to ensure that these benefits to cardiac vascularity are retained even when the negative vascular effects of doxorubicin are confirmed.

In line with the similar cardiac capillary densities, there were no significant differences in the cardiac VEGF-A and HIF1-α levels between the groups. Despite no change in the cardiac HIF1-α level, there was a significant increase in its downstream target, the cardiac LDH activity, in the exercised mice, but this was not the case for CS activity. This suggests that the increase in LDH activity was induced by another route. Increased LDH activity in the exercised animals suggests increased anaerobic energy production capacity to meet the increased energy requirements, therefore suggesting that the wheel running exercise may have had a beneficial effect on cardiac energy production. It could also be that the increased LDH activity represents increased LDHB activity rather than LDHA, suggesting that the heart adapts its metabolism to use lactate produced by the muscles as a source of energy. Previously, at least ventricular citrate synthase activity has been shown to be inhibited by doxorubicin (Yao et al., 2011). Despite the positive changes in the energy production capacity, the exercise did not affect the cardiac oxidative damage to lipids nor increased the activity of the antioxidative enzyme catalase in chemotherapy-treated mice. We, however, cannot exclude the possibility that other antioxidative enzymes might have been affected by the exercise. Previously, treadmill exercise (45 min/day x 5/week, 12 m/min) done for 2 weeks has been shown to reduce the production of reactive oxidative species and preserve the cardiac function both in immunocompetent and immunocompromised tumor-free juvenile mice receiving doxorubicin treatment (Wang et al., 2021). Doxorubicin has also been shown to reduce cardiac catalase activity with 9-week treadmill running exercise performed concomitantly with doxorubicin treatment, increasing catalase activity and the activity of other antioxidative enzymes in tumor-free female rats (Sequeira et al., 2021). However, another study on female mice using an 8-week treadmill exercise protocol with a slightly different intensity and treatment protocol found no such effect on catalase activity (Dolinsky et al., 2013), showing the importance of the differences in the exercise regimen on the cardiac benefits. Nevertheless, preclinical studies, in general, support that both long-term and even short-term voluntary and forced exercise can benefit the heart during doxorubicin treatment (Dozic et al., 2023). However, the precise effects can vary depending on the mode and intensity of exercise. In the current study, we used voluntary running wheel exercise to allow a more natural running behavior with less stress to the animal and a comfortable intensity of exercise at the cost of not being able to control the exercise intensity and amount. It is possible that the benefits of exercise are more apparent when the cardiotoxic effects of doxorubicin increase or when exercise is performed for a longer duration or at a higher intensity. Lastly, it must be noted that due to the lack of a healthy control group, it is not possible to say whether the treatment regimen itself was hindering the cardiovascular benefits of exercise or whether the oxidative damage to lipids was altered compared to the healthy state. It has been previously shown that doxorubicin treatment of mammary tumor-bearing mice with cardiotoxic effects does not always increase cardiac markers of oxidative damage (Cheong et al., 2021). However, previously, the cumulative dosage of 15 mg/kg of doxorubicin with varying dosing regimens has been shown to induce reduced fractional shortening, cardiac hypertrophy or atrophy, and fibrosis, as well as increased cardiac oxidative stress and apoptosis in male mice (Sahu et al., 2016; Pillai et al., 2017), while not many studies have tested this dosage on female mice (Podyacheva et al., 2021). Nonetheless, it can still be deduced whether this level of physical activity would show benefits for cardiovascular function during doxorubicin treatment of female mice with breast cancer at this stage of cumulative dosage. Overall, our results suggest that even light exercise (voluntary running wheel exercise) performed right after tumor inoculation and concomitantly with doxorubicin treatment can yield some cardiovascular benefits at this intensity and duration. Whether the benefits of voluntary running wheel exercise will be retained with a longer duration of exercise and longer treatment with increasing cardiotoxic effects still needs to be evaluated with this tumor-bearing model.

### 4.2 Subcutaneous mammary tumor growth in response to physical activity

The second aim of this study was to investigate whether short-term voluntary wheel-running exercise and chemotherapy treatment together could enhance tumor size reduction. In this study, all animals began to grow tumors after a week of inoculating tumor cells subcutaneously, and no animal had to be removed from the study due to decreased welfare or weight loss, but two animals had to be euthanized prior to being given the final dose of chemotherapy due to ulceration of the tumor and the tumor size. The used cancer cells originate from the breast cancer tumors of the transgenic MMTV-PYMT mice, and in this model, a high proportion of tumor cancer cells lose their progesterone and ER receptors at the late stages of the tumor development (Lin et al., 2003). A different cell line isolated from the same model has been shown to spread to the subcutis after inoculation to the mammary fat pad (Werbeck et al., 2014). The inoculated cells in the current study formed fast growing tumors, creating a realistic target for chemotherapy, and by inoculating subcutaneously, they can be considered to mimic the growth of the tumor after spreading to nearby tissues from the mammary tissues (Zeng et al., 2020). The fast growth of the I3TC tumors was even more apparent in the mice without doxorubicin treatment, as shown in Supplementary Material. The results revealed that the group had a significant effect on the tumor volumes over time; exercised animals seemed to have smaller tumor volumes compared to the animals in the control group. This was seen especially toward the end of the experiment. Despite the apparent reduction in the tumor growth rate and the differing tumor volumes over time between the groups, there was no difference between the final tumor volumes at the end of the study. However, there was high individual variability in tumor growth, which might make it hard to detect more modest effects of exercise on tumor growth. The tumor inoculation process itself can induce differences in the tumor models by altering, for example, the exact cell number injected, with the phenotypic variability of the individuals further effecting the tumor growth. It also needs to be pointed out that we did not use the orthotopic tumor model since the location of the mammary tumor might have prevented the animals from running. In future studies, it should be investigated whether exercise can have positive effects on reducing tumor volume in orthotopic models as well. Nevertheless, our results suggest that this amount of exercise does not hinder the efficacy of doxorubicin treatment but may possibly have a beneficial effect on the treatment efficacy when exercise is started right after cancer cell inoculation and continued concomitantly with the doxorubicin treatment. This might, in clinical setting, compare to starting the exercise after diagnosis prior to starting chemotherapy treatment and continuing exercise after the start of the treatment.

Exercise increased the number of positive cells for the apoptosis marker cleaved-cas3 in the tumors, further supporting our finding that tumor growth was reduced in exercising animals. However, there seemed to be no difference in the proliferation of the tumor cells (a similar number of cells positive for ki67). This suggests that tumor growth was not altered by exercise via changes in tumor cell proliferation but rather via increased tumor cell death. There was no evidence suggesting that exercise improved the doxorubicin efficacy on the tumors via improved drug delivery as the capillary density, blood vessel maturation, and levels of HIF1-α and VEGF-A were similar between the groups. However, verifying this direct blood flow measurement would be needed in the future. In subcutaneous melanoma and PDAC-4662 pancreatic cancer, exercise has been shown to improve drug delivery and treatment efficacy of chemotherapy without changes in capillary density or tumor blood vessel maturation, with a greater effect on blood vessel normalization (Schadler et al., 2016). However, exercise has been shown to improve tumor blood flow via increased capillary density in mice without chemotherapeutic treatment that had orthotopic human mammary tumor (MDA-MB-231) xenografts (Jones et al., 2010). One study found that exercise induced vessel normalization and decreased hypoxemia without any effect on vessel density in mouse mammary tumors without chemotherapeutic treatment (Gomes-Santos et al., 2021b). Similarly, another study showed that exercise during doxorubicin treatment of mouse breast cancer could also decrease tumor hypoxemia, as evidenced by reduced HIF1-α and HIF2-α, and enhance the antitumor effect when tumor growth was compared to untreated animals (Wakefield et al., 2021). Contradictory to these findings, treadmill exercise did not improve the doxorubicin treatment efficacy in mice with subcutaneous human mammary tumor (MDA-MB-231) xenografts (Jones et al., 2005). Similarly, it has been found that exercise had no effect on tumor growth, tumor blood vessel density, number of perfused blood vessels, and tumor cell proliferation in treatment-naïve immunocompetent mouse models of orthotopic E0771 breast cancer (Buss et al., 2020). These differences in study outcomes are likely due to differences in the form of exercise and its intensity, as well as differences in the treatment plan, tumor model, and the mouse model used. Differences in study results could partially also be affected by the method used in capillary density calculation. Several studies, including our study, calculate the number of capillaries from randomly chosen areas (Schadler et al., 2016; Buss et al., 2020) or from random entire cross sections of the tumor, from which the average for total tissue is estimated. However, a tumor may contain capillary hotspots, and selecting areas randomly can, by chance, skew the results if hotspots are included or not. Calculating vessel density from the entire tumor, assuming that no tissue from the section is lost during processing, is more trustworthy but much more cumbersome to analyze and consumes the whole tumor. Additionally, the lack of chemotherapeutic treatment may affect the outcome regarding the effect of exercise on treatment efficacy because there is some evidence that chemotherapeutic treatment itself affects tumor vasculature (Fung et al., 2015). In this study, the activities of the metabolic enzymes citrate synthase and lactate dehydrogenase, which are closely linked to blood flow and oxygen delivery to tissue, were similar between the two groups. Therefore, it seems that exercise acts via some other mechanism than those measured in this study, with one of them possibly being via immune system cells. It has been shown previously that exercise alone does reduce the MMTV-PyMT-tumor growth, the model from which the cancer cells in the current study were derived, via its effects on the immune system cells (Rundqvist et al., 2020). For example, exercise has been shown to increase the natural killer cell infiltration to the tumor site via exercise-induced epinephrine and IL6 secretion (Pedersen et al., 2016). However, we did not measure this or other effects on the immune system cells and, therefore, cannot speculate whether this contributed to the increased tumor apoptosis. In the current study, measuring immune cell infiltration was not possible as it is sensitive to the timing of the exercise bout, which cannot be controlled when using voluntary running exercise (Koivula et al., 2023). Therefore, further investigations are needed regarding possible changes in immune cell infiltration and subcutaneous mammary tumor blood flow, with *in vivo* imaging being the best way to confirm whether blood flow to the subcutaneous mammary tumors during rest is improved as a result of exercise training. Subcutaneous breast cancer tumors tend to grow faster than orthotopic tumors (O’Keefe et al., 1993), and the tumor microenvironment is different than it would be for the primary tumor, but subcutaneous models can, to some extent, model the secondary tumor growth and account for the effect of some of the tumor-secreted factors. Nonetheless, the tumor location can affect the outcome of the study, especially the findings regarding the tumor microenvironment, and therefore, further studies with orthotopic models are needed to confirm the findings of this study.

### 4.3 Study limitations

The lack of tumor-free control or untreated mammary tumor-bearing mice and the use of the non-orthotopic tumor model should be acknowledged as limitations to this study. However, to mitigate this limitation, we added a comparison to an untreated control done at a later time point in the supplements. A lack of healthy and untreated controls and the low n-number were maintained to adhere to the principle of the three Rs and minimize the number of animals used. The n-number and leaving out the healthy and untreated group was considered sufficient to address whether this amount of exercise could benefit the heart and affect tumor growth. However, the high variability of the tumor growth and mouse running activity with the low n-number together caused the statistical power to be lower than desired in some of the statistical tests done, and therefore, the negative results regarding tumor growth and running activity must be interpreted with caution. We also cannot exclude the effects of animal estrous cycles on tumor growth as this was not monitored (Wood and Hrushesky, 2005). Another limitation to the study arises from the co-housing of two mice per cage, preventing an accurate analysis of individual mouse running activity. Similarly, the food intake of the animals could not be monitored for this reason, which could potentially affect the results. However, this was considered a necessary compromise to improve animal welfare as the animal activities could still be estimated with reasonable accuracy. The lack of orthotopic tumor model is also a study limitation due to the tumor microenvironment being different for the breast cancer cells. The orthotopic model was not used to avoid the risk of interfering with the ability of the animals to remain physically active due to the location of the orthotopic mammary tumors and their possibly large final sizes. Previously, Met-1 cells isolated from the MMTV-PyMT cancer model, which are similar in origin to the cells used in this study, have been shown to grow significantly faster subcutaneously compared to the mammary fat pad, although the injected cell number likely plays a significant role in tumor growth as well (Werbeck et al., 2014). Despite this, the orthotopic and subcutaneous locations behave similarly when it comes to the formation of metastasis, showing that Met-1 cancer cells are not likely to metastasize from either of these locations (Werbeck et al., 2014). The commonly used method of calculating average capillary density from random smaller areas of the tumor can also be considered a limitation as possible capillary hotspots in tumor could potentially skew the data.

### 4.4 Conclusions

In conclusion, voluntary running wheel exercise done concomitantly with treatment may have beneficial effects on treatment efficacy by reducing the tumor growth via increasing tumor cell apoptosis and have modest beneficial effects on the heart via the increased number of capillaries per cell and higher LDH activity. The intraperitoneal dosing of 5 mg/kg/week of doxorubicin up to three times allowed FVB-mice bearing subcutaneous mammary tumors to stay physically active and perform running wheel exercise with no significant weight loss, although their natural weight gain compared to the exercising animals was blunted. Further studies are needed on the molecular background of doxorubicin-induced cardiac alterations and the effects of exercise intervention on the heart and the tumor in mammary tumor-bearing mice. Further studies are also needed using, for example, the orthotopic model of breast cancer to better translate the results to a clinical setting, although this may prove challenging if the location of the tumor affects the running activity.

## Data Availability

The original contributions presented in the study are included in the article/Supplementary Material; further inquiries can be directed to the corresponding author.
