# Supplementary material for: Voluntary wheel running reduces tumor growth and increases capillarity in the heart during doxorubicin chemotherapy in a murine model of breast cancer
Source: Front Physiol. 2024 Apr 25;15:1347347. doi: 10.3389/fphys.2024.1347347 (PMC11079236; doi:10.3389/fphys.2024.1347347)
Supplement: Supplementary file 1 [file DataSheet1.pdf]

Supplementary Material

1 Supplementary Data

Supplementary Data 1. Macro scripts for analysis of tumor blood vessel maturation, tumor cas3 and staining k67 staining.

Macro script for processing the images for the analysis of tumor blood vessel maturation.

Green channel image processing for background removal:

```
run("Duplicate...", "title=original");
run("Duplicate...", "title=copy");
run("Subtract Background...", "rolling=50 create sliding");
imageCalculator("Subtract create 32-bit", "original","copy");
run("6 shades");
```

Red channel image processing for background removal:

```
run("Retinex", "level=Low scale=250 scale_division=8 dynamic=4");
```

Macro script for tumor cas3 analysis on ImageJ. Requires installation of Color deconvolution 2:

Landini G, Martinelli G, Piccinini F. Colour Deconvolution – stain unmixing in histological imaging. Bioinformatics 2020, https://doi.org/10.1093/bioinformatics/btaa847
Ruifrok AC, Johnston DA. Quantification of histochemical staining by color deconvolution. Analytical and Quantitative Cytology and Histology 23: 291-299, 2001.

```
//choose input and output directory and allow batch processing
dir1 = getDirectory("Choose Source Directory ");
dir2 = getDirectory("Choose Destination Directory ");
list = getFileList(dir1);
setBatchMode(true);
for (i=0; i<list.length; i++) {
 showProgress(i+1, list.length);
 open(dir1+list[i]);
//do user defined decolor convolution for DAP, the color values need to be defined separately for
each stain
run("Colour Deconvolution2", "vectors=[User values] output=[8bit_Transmittance] simulated cross
[r1]=0.36004835 [g1]=0.5594055 [b1]=0.7466128 [r2]=0.5510393 [g2]=0.56888634
[b2]=0.61051124 [r3]=0.5020557 [g3]=0.63004726 [b3]=0.5924361");
//close files we do not need, the color images 3 and 2, last active image remaining is the color1 for
DAB
close();
close();
//Next run threshold and covert image to mask and edit the mask
run("Threshold...");
//Below values must be edited to suit each staining/dataset
```

```
setThreshold(0, 70);
run("Convert to Mask");
run("Erode");
run("Dilate");
run("Dilate");
run("Erode");
run("Watershed");
run("Erode");
run("Watershed");
//Next analyze particles, display summarize and add new lines for all the counted particles
//The particle size should be adjusted according to dataset
run("Analyze Particles...", "size=20-Infinity display summarize add");
Imgname = getTitle;
roiManager("Save", dir2 + Imgname + ".zip");
roiManager("reset");
//Note this does not save the color deconvolution result image or the mask
//This saves only ROIS that can be applied on top of the cropped images to check accuracy
}
```

Macro script for tumor ki67 analysis on ImageJ. Requires installation of Colon deconvolution 2:

```
//choose input and output directory and allow batch processing
dir1 = getDirectory("Choose Source Directory ");
dir2 = getDirectory("Choose Destination Directory ");
list = getFileList(dir1);
setBatchMode(true);
for (i=0; i<list.length; i++) {
 showProgress(i+1, list.length);
 open(dir1+list[i]);
 //reduce noise next via blurring
 run("Gaussian Blur...", "sigma=0.55");
//do user defined decolor convolution for DAB, the color values need to be defined separately for
each stain
run("Colour Deconvolution2", "vectors=[User values] output=8bit_Transmittance simulated cross
hide [r1]=0.4318502607617876 [g1]=0.5705871783319327 [b1]=0.698523889500695
[r2]=0.5872162354922159 [g2]=0.5724373053615174 [b2]=0.5722697128145044
[r3]=0.5474160033763609 [g3]=0.603950887814577 [b3]=0.5792918473062059");
//close extra windows, the color images 3 and 2, last active image remaining is the color1 for DAB
close();
close();
//reduce background
run("Subtract Background...", "rolling=2400 light");
//Next run threshold and covert image to mask and edit the mask
run("Threshold...");
//Below values must be edited to suit each staining/dataset
setThreshold(0, 37, "Otsu no-reset");
```

```
run("Convert to Mask");
run("Dilate");
run("Close-");
("Fill Holes");
run("Watershed");
run("Erode");
run("Dilate");
run("Dilate");
("Fill Holes");
run("Watershed");
run("Erode");
run("Dilate");
run("Dilate");
run("Dilate");
run("Dilate");
run("Dilate");
("Fill Holes");
run("Watershed");
run("Erode");
//Next analyze particles, display summarize and add new lines for all the counted particles
//The particle size should be adjusted according to dataset
run("Analyze Particles...", "size=40-Infinity display summarize add");
Imgname = getTitle;
roiManager("Save", dir2 + Imgname + ".zip");
roiManager("reset");
//Note this does not save the color deconvolution result image or the mask
//This saves only ROIS that can be applied on top of the cropped images to check accuracy
}
```

2 Supplementary Figures and Tables

Supplementary Table 1. The raw measurements showing the length (L, mm), width (w, mm) and volume (V, mm3) per day of the tumors for every mouse used in the present study.

**Days after inoculation**			*3*			*7*			*11*			*14*			*16*			*18*			21			23			25			28			29		
**Cage**	**Animal**	**Group**	**L**	**W**	***V***	**L**	**W**	***V***	**L**	**W**	***V***	**L**	**W**	***V***	**L**	**W**	***V***	**L**	**W**	***V***	**L**	**W**	***V***	**L**	**W**	***V***	**L**	**W**	***V***	**L**	**W**	***V***	**L**	**W**	***V***
1	1	Exercise	1	1	0.5	4	3	18.8	4	4	33.5	5	4	41.9	5	5	65.4	6	5	78.5	8	6	150.7	7	6	131.9	7	7	179.5	7	6	131.9	6	6	113.0
	2	Exercise	1	1	0.5	4	3	18.8	4	3	18.8	4	3	18.8	3	3	14.1	3	3	14.1	6	5	78.5	7	6	131.9	7	7	179.5	7	6	131.9	7	5	91.6
2	3	Control	1	1	0.5	5	4	41.9	5	4	41.9	6	5	78.5	6	4	50.2	6	6	113.0	8	7	205.1	8	6	150.7	7	6	131.9	7	6	131.9	6	6	113.0
	4	Control	1	1	0.5	4	4	33.5	5	5	65.4	7	6	131.9	8	7	205.1	9	8	301.4	9	7	230.8	8	6	150.7	8	7	205.1	8	7	205.1	8	8	267.9
3	5	Exercise	2	1	1.0	6	5	78.5	8	6	150.7	9	7	230.8	9	7	230.8	8	7	205.1	6	6	113.0	[redacted]											
	6	Exercise	1	1	0.5	4	4	33.5	5	5	65.4	9	6	169.6	9	7	230.8	10	7	256.4	11	9	466.3	9	7	230.8	10	7	256.4	10	8	334.9	[redacted]		
4	7	Control	1	1	0.5	4	4	33.5	5	5	65.4	7	6	131.9	7	7	179.5	9	8	301.4	6	6	113.0	6	5	78.5	5	5	65.4	5	4	41.9	5	4	41.9
	8	Control	1	1	0.5	3	3	14.1	4	4	33.5	5	4	41.9	6	5	78.5	6	6	113.0	6	6	113.0	9	8	301.4	7	7	179.5	7	7	179.5	8	7	205.1
5	9	Exercise	1	1	0.5	5	3	23.6	6	5	78.5	8	7	205.1	8	6	150.7	8	6	150.7	8	6	150.7	7	7	179.5	7	7	179.5	6	5	78.5	5	5	65.4
	10	Exercise	1	1	0.5	4	3	18.8	6	5	78.5	7	7	179.5	8	7	205.1	9	9	381.5	10	9	423.9	10	8	334.9	9	8	301.4	8	7	205.1	7	6	131.9
6	11	Control	1	1	0.5	5	4	41.9	6	5	78.5	7	5	91.6	7	5	91.6	6	6	113.0	7	5	91.6	7	6	131.9	7	6	131.9	8	6	150.7	6	6	113.0
	12	Control	1	1	0.5	3	3	14.1	6	4	50.2	9	5	117.8	10	9	423.9	12	10	628.0	16	11	1013.2	[redacted]											
7	13	Exercise	~0.5		0.1	2	2	4.2	2	2	4.2	6	5	78.5	8	8	267.9	9	8	301.4	10	10	523.3	10	10	523.3	10	9	423.9	10	7	256.4	9	7	230.8
	14	Exercise	1	1	0.5	5	4	41.9	6	5	78.5	7	5	91.6	8	6	150.7	8	7	205.1	10	7	256.4	9	8	301.4	8	8	267.9	8	7	205.1	8	7	205.1
8	15	Control	~0.5		0.1	4	4	33.5	8	7	205.1	9	8	301.4	9	8	301.4	10	8	334.9	12	10	628.0	11	10	575.7	12	11	759.9	[redacted]					
	16	Control	1	1	0.5	4	4	33.5	6	5	78.5	8	6	150.7	8	7	205.1	10	9	423.9	11	8	368.4	10	10	523.3	10	9	423.9	11	9	466.3	11	8	368.4

X1 = euthanized because of wound on tumor. X2 = euthanized. X3 = euthanized because of tumor size. X4 = euthanized early because would reach end point during weekend.

Table 2. The products and the protocol of the histological staining of paraffin embedded mouse hearts and mammary tumors.

Step	Heart capillary stain	Tumor capillary stain	Tumor blood vessel maturation	Tumor cleaved cas3	Tumor ki67
Antigen retrieval	IHC antigen retrieval low pH (eBioscience, Cat 00-4955-58)	IHC antigen retrieval low pH (eBioscience, Cat 00-4955-58)	IHC antigen retrieval low pH (eBioscience, Cat 00-4955-58)	IHC antigen retrieval high pH (eBioscience, Cat 00-4956-58)	IHC antigen retrieval low pH (eBioscience, Cat 00-4955-58)
Peroxidase quenching	3 % H_2O_2 10 min	3 % H_2O_2 10 min	None	3 % H_2O_2 10 min	3 % H_2O_2 10 min
Blocking	1 % BSA in PBS, 30 min at a room temperature.				
Primary antibody	Goat anti-mouse anti-Podocalyxin (1:300, R&D systems AF1556)	Goat anti-mouse anti-Podocalyxin (1:300, R&D systems AF1556)	Goat anti-mouse anti-Podocalyxin (1:300, R&D systems AF1556) 2h at RT	Rabbit anti human/mouse anti-caspase 3 (1:200, R&D Systems AF835)	Rabbit anti-Ki-67 (1:200 Sigma Aldrich ab9260)
Secondary antibody	Anti-goat biotinylated antibody 1:500, 45 min RT	Anti-goat biotinylated antibody 1:500, 45 min RT	Anti-goat AlFl.488 (1:100, LifeTech A11055) overnight +4 °C	Anti-rabbit biotinylated antibody (1:500, 45 min RT)	Anti-rabbit biotinylated antibody (1:500, 45 min RT)
2nd primary antibody	-	-	Mouse monoclonal anti-SMA –Cy3 (1:100, Sigma C6198) 2 h RT	-	-
Peroxidase staining	ABCPeroxidase Staining Kit (Thermo Fisher Scientific, Cat 32020) in PBST, 45 min RT	ABCPeroxidase Staining Kit (Thermo Fisher Scientific, Cat 32020) in PBST, 45 min RT	-	ABCPeroxidase Staining Kit (Thermo Fisher Scientific, Cat 32020) in PBST, 45 min RT	ABCPeroxidase Staining Kit (Thermo Fisher Scientific, Cat 32020) in PBST, 45 min RT
Peroxidase substrate	DAP substrate kit (Thermo Fisher Scientific Cat 34065) 6 min RT	DAP substrate kit (Thermo Fisher Scientific Cat 34065) 6 min RT	-	DAP substrate kit (Thermo Fisher Scientific Cat 34065) 2 min RT	DAP substrate kit (Thermo Fisher Scientific Cat 34065) 3 min RT
Counterstain	Mayer's Hematoxylin (Sigma, MHS16-500mL)	Mayer's Hematoxylin (Sigma, MHS16-500mL)	DAPI in mounting media	Mayer's Hematoxylin (Sigma, MHS16-500mL)	Mayer's Hematoxylin (Sigma, MHS16-500mL)

Antigen retrieval was performed by boiling samples 10 minutes followed by cooling them to room temperature. All antibodies were diluted in 0.5 % of blocking solution in PBST and the primary antibody incubations were done overnight at +4 °C if not stated otherwise. All steps are followed by 3 x 5 min PBST (PBS + 0.1 % Tween-20) washes, except for DAB reaction, which was followed by rinsing with PBS and double distilled H_2O, and the hematoxylin step, which was followed by 10 min ddH_2O wash.

Table 3. The products used for the tissue homogenization and western blot.

Homogenization solution	62.5 mM Tris-HCl, 1 µg.mL^{-1} leupeptin, 1 µg.mL^{-1} pepstatin, 1 mM phenylmethylsulfonyl fluoride
Laemmli solution	625 mM Tris pH6.8, 20 % glycerol, 2 % SDS, 0.025 % bromophenol blue, β-mercaptoethanol 5 %

All of the tissues were kept in cold and homogenized 1:6 mg µl-1 of homogenization solution, using two steel beads and TissueLyser (Qiagen, Cat. 85220, Germany) 2 x 2 min at a frequency 30 s-1 in +4 °C. The homogenates were centrifuged at 5000 g for 5 minutes at 4 °C and the supernatant was collected for determination of the protein content using the Bicinchoninic acid assay (BCA, Pierce®, Thermoscientific, USA). The samples were diluted 1:1 in Laemmli solution and heat treated in 70 °C for 7 minutes after which they were stored in -80 °C.

2.1 Supplementary Figures

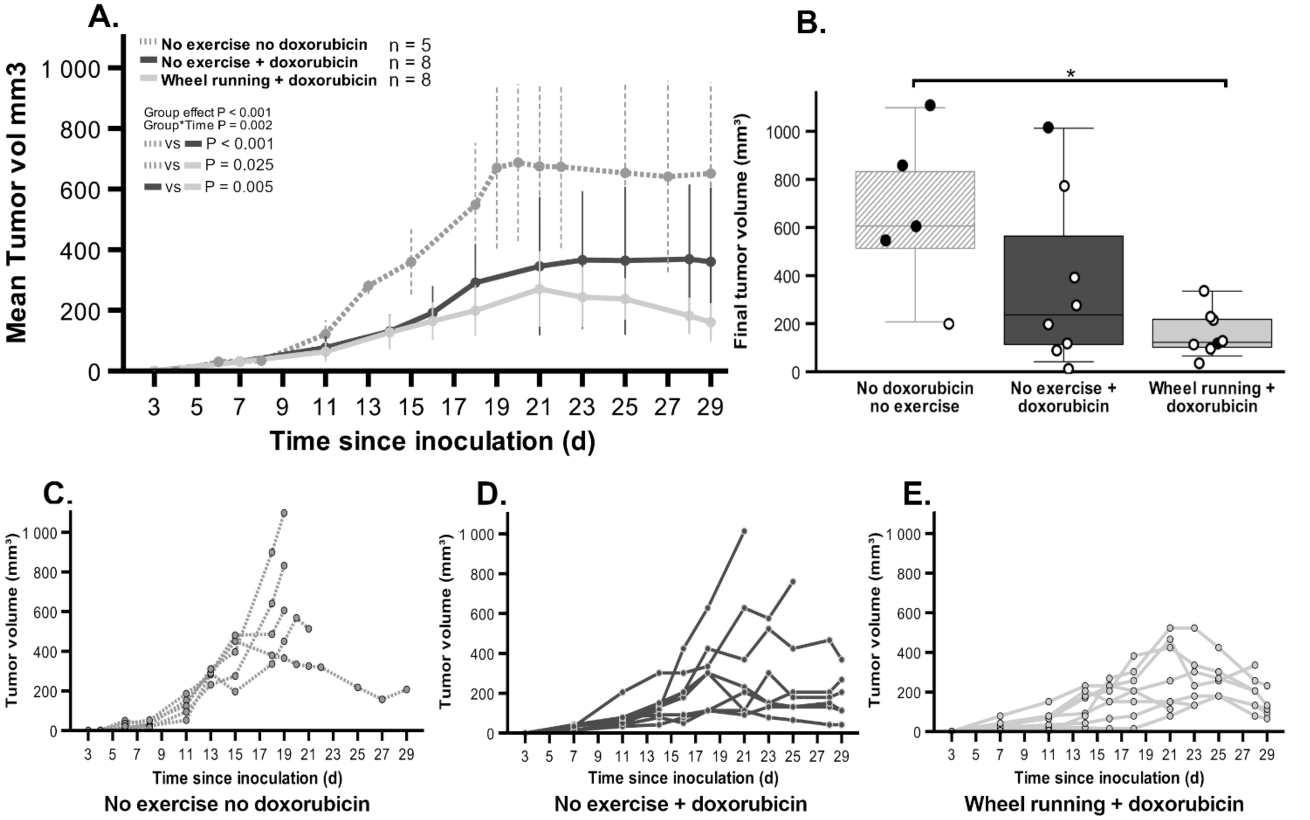

Supplementary Figure 1. The subcutaneous breast cancer tumor growth over time and the end of study in comparison to mice with no doxorubicin treatment. From the individual tumor growths, it is apparent that the untreated tumors reached the end point criteria much faster than treated tumors and the supplementary figure 1A shows that on average the untreated animals had significantly larger tumor volumes overtime. The no exercise no doxorubicin treatment group data is obtained from FVB-mice that were inoculated subcutaneously with the 13TC breast cancer cells at a different time and in different facility (ethical license 26508-2021 appoved by southern Finland regional administration agency ESAVI). The tumor growth in the three groups of mice overtime (A) is presented with the P values for the linear regression test comparing the groups for fixed main effects of group, time and their interaction with a Holm Sidak post hoc test used for between groups comparisons. The final tumor volumes in each group (B) are presented with the datapoints shown in black if the animal had to be euthanized before the last experiment day 29. The individual tumor growths in each group (C-E). One-way Anova post hoc test *P<0.05.

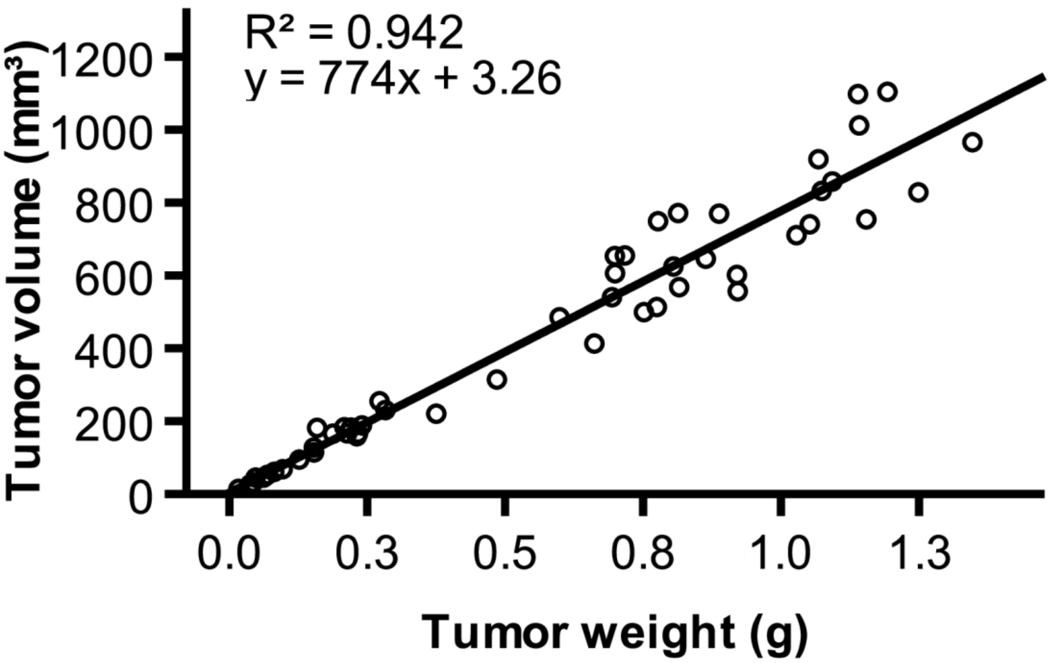

Supplementary Figure 2. The linear correlation of tumor volume and weight of tumors originating from subcutaneously injected I3TC breast cancer cells. The data presented is from our other unpublished study not part of the current study that was also using FVB/N mice. Tumor volumes presented were calculated using the formula: width*length*depth*3.14/6, with the tumor width, depth and length being measured upon tumor extraction using calipers. The linear function presented was used to estimate the tumor weights of the current study as the tumors in the current study could not be weighed at the time of the study due to insufficient accuracy of the scale.

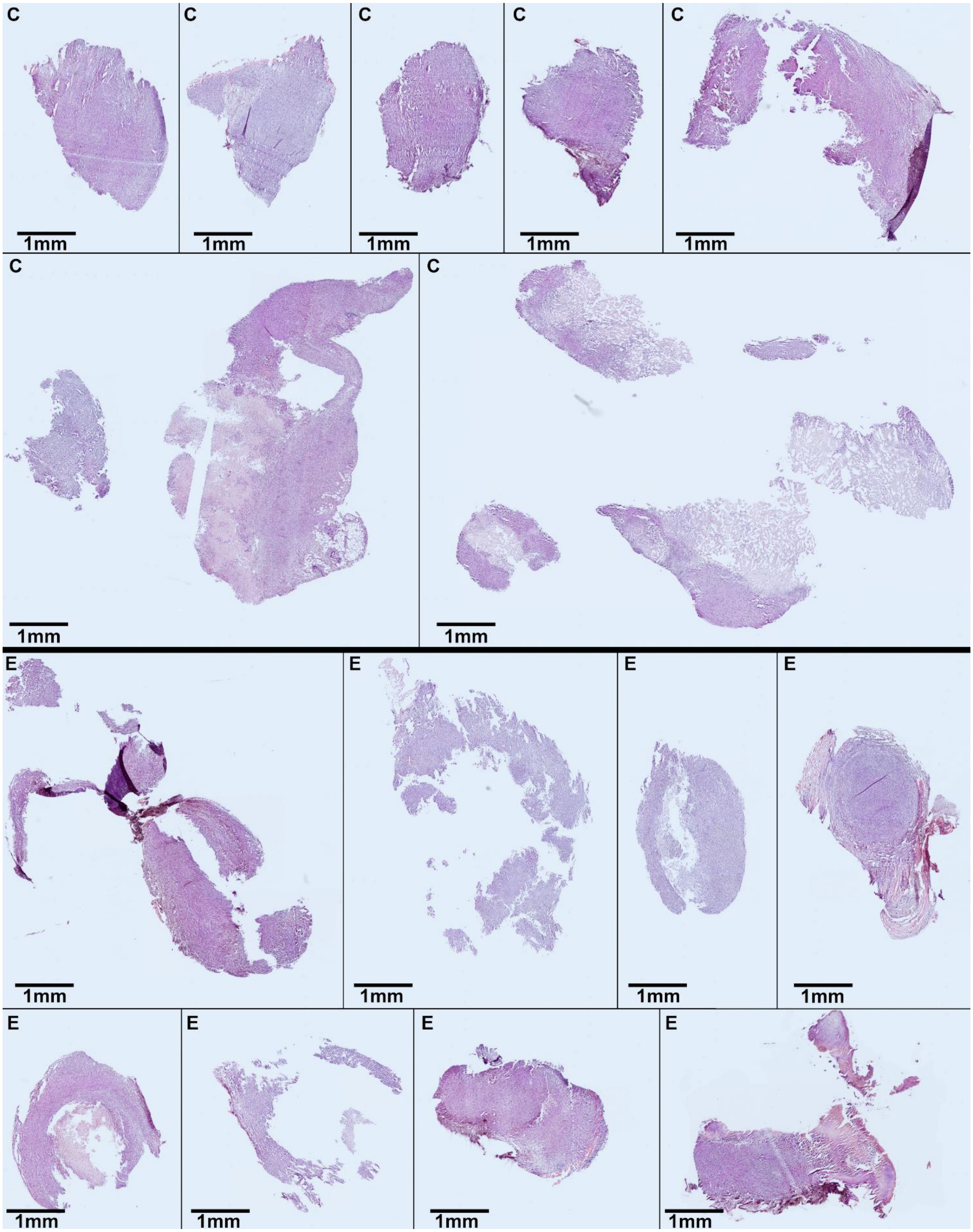

Supplementary Figure 3. Hematoxylin eosin stains of the subcutaneous I3TC breast cancer tumors of all animals which were treated with doxorubicin. The E and C within each image represents the group, exercise or no-exercise control.

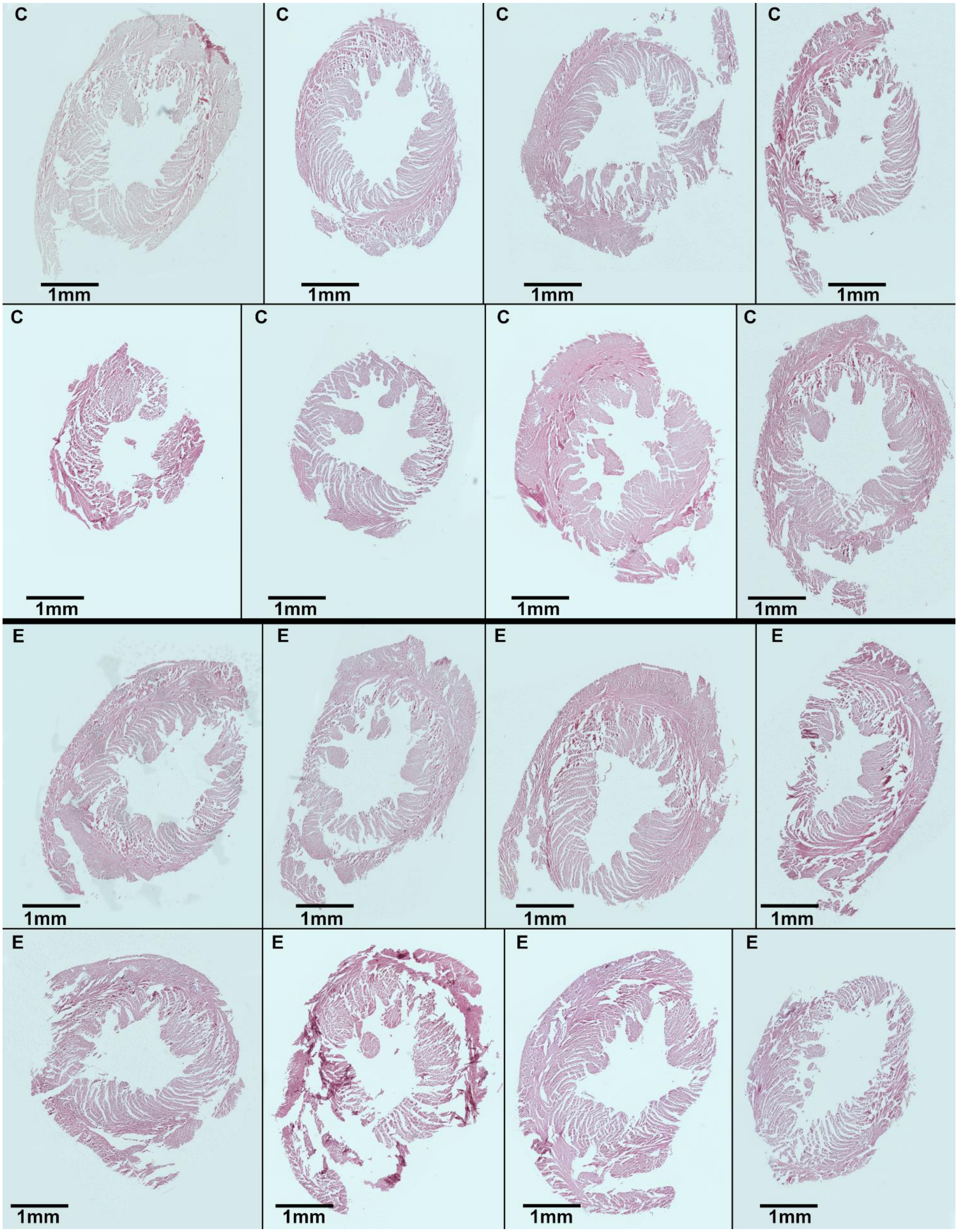

Supplementary Figure 4. Hematoxylin eosin stains of transversal heart sections of doxorubicin treated subcutaneous breast cancer bearing mice. The group is indicated with E (exercise) or C (no-exercise control.)

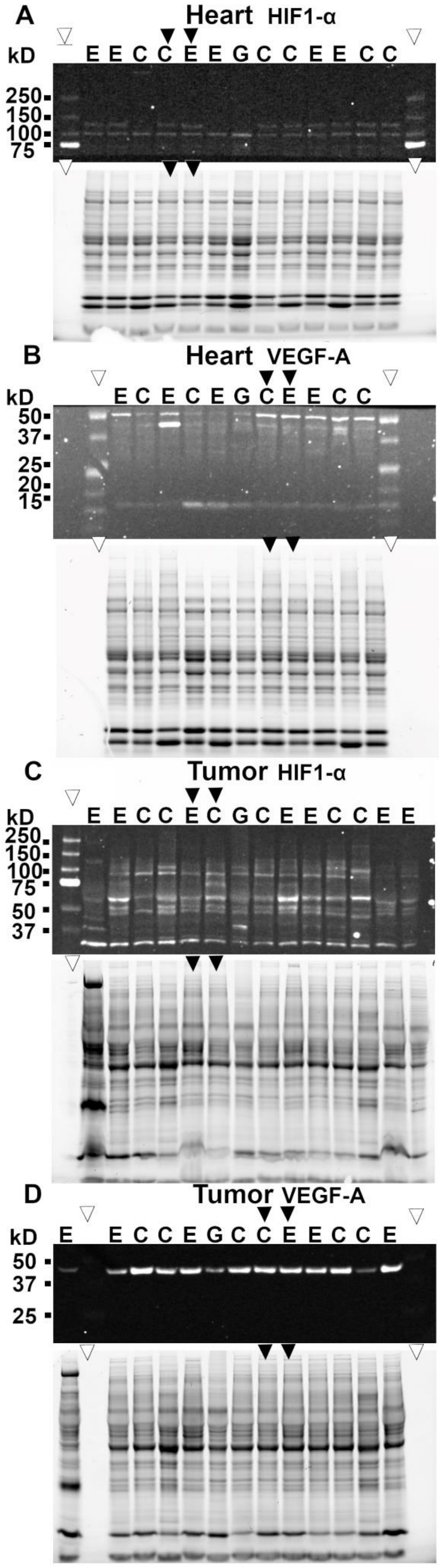

Supplementary Figure 5. Exemplary whole western blot membranes and gels. The heart HIF1-α membrane (A) and VEGF-A membranes (B) are shown with the corresponding gel image below of the overall protein amount that was used as a loading control. The Tumor HIF1-α membrane (C) and VEGF-A membrane (D) are shown with the corresponding gel images of the overall protein amounts. The samples presented in the images of the manuscript are indicated by black arrows while the protein ladders are indicated by white arrows. Sample identities are shown with E (exercise), C (no exercise control) or with G (gel control; for calculating inter-gel variation). All bands that were improperly stained were excluded and re-done.
